# Cost-effectiveness of TB diagnostic technologies in Ethiopia: a modelling study

**DOI:** 10.1186/s12962-024-00544-1

**Published:** 2024-05-21

**Authors:** Lelisa Fekadu Assebe, Andargachew Kumsa Erena, Lemmessa Fikadu, Bizuneh Alemu, Yirgalem Shibiru Baruda, Boshen Jiao

**Affiliations:** 1 Department of Global Public Health and Primary Care, Faculty of Medicine, University of Bergen, Bergen, Norway; 2 Department of Global Health and Population, Harvard T.H. Chan School of Public Health, Boston, MA USA; 3 Disease Prevention and Control Program, Ministry of Health, Addis Ababa, Ethiopia; 4 Health system strengthening through Performance Based Financing Project, Cordaid, Bahir dar, Ethiopia; 5 Department of Health Promotion and disease prevention, Oromia Regional Health Bureau, Addis Ababa, Ethiopia; 6 Department of Global Health, Faculty of Health, Medicine and Life Sciences, Maastricht University, Maastricht, Netherlands

**Keywords:** Tuberculosis, Cost-effectiveness, Microscopy, Rapid TB diagnostics, Ethiopia

## Abstract

**Background:**

Tuberculosis (TB) is a major threat to public health, particularly in countries where the disease is highly prevalent, such as Ethiopia. Early diagnosis and treatment are the main components of TB prevention and control. Although the national TB guideline recommends the primary use of rapid TB diagnostics whenever feasible, there is limited evidence available that assess the efficiency of deploying various diagnostic tools in the country. Hence, this study aims to evaluate the cost-effectiveness of rapid TB/MDR-TB diagnostic tools in Ethiopia.

**Methods:**

A hybrid Markov model for a hypothetical adult cohort of presumptive TB cases was constructed. The following TB diagnostic tools were evaluated: X-pert MTB/RIF, Truenat, chest X-ray screening followed by an X-pert MTB/RIF, TB-LAMP, and smear microscopy. Cost-effectiveness was determined based on incremental costs ($) per Disability-adjusted Life Years (DALY) averted, using a threshold of one times Gross Domestic Product (GDP) per capita ($856). Data on starting and transition probabilities, costs, and health state utilities were derived from secondary sources. The analysis is conducted from the health system perspective, and a probabilistic sensitivity analysis is performed.

**Result:**

The incremental cost-effectiveness ratio for X-pert MTB/RIF, compared to the next best alternative, is $276 per DALY averted, making it a highly cost-effective diagnostic tool. Additionally, chest X-ray screening followed an X-pert MTB/RIF test is less cost-effective, with an ICER of $1666 per DALY averted. Introducing X-pert MTB/RIF testing would enhance TB detection and prevent 9600 DALYs in a cohort of 10,000 TB patients, with a total cost of $3,816,000.

**Conclusion:**

The X-pert MTB/RIF test is the most cost-effective diagnostic tool compared to other alternatives. The use of this diagnostic tool improves the early detection and treatment of TB cases. Increased funding for this diagnostic tool will enhance access, reduce the TB detection gaps, and improve treatment outcomes.

## Background

Tuberculosis (TB) is a major cause of death worldwide, with estimated cases and deaths ranging from 10.6 to 1.3 million in 2022 [1]. The burden of TB is closely linked to socioeconomic inequality and poverty [2]. Globally, the incidence and mortality of TB decreased by 9 and 19% between 2015 and 2022, respectively, which falls short of the targets set by the “End TB strategy” to be met by 2025. The End TB strategy has been put into action over the past years, with the goal of ending TB as a public health threat in 2035 [3]. The strategy provides a systemic response to address the adverse health and economic impact of TB through the implementation of the elements under the following pillars: integrated, patient-centred care and prevention; bold policies and support systems; intensified research and innovation [3–5]. Although countries aspire to end TB by 2035, progress has lagged considerably behind the “End TB strategy” goals [1].

In Ethiopia, the burden of TB has consistently declined by 7–8% annually, reaching a TB incidence of 126 per 100,000, respectively, in 2022 [1, 6, 7]. The adoption of innovative technology, including the rollout of rapid diagnostics for TB (e.g., X-pert MTB/RIF, LED microscopy, digital X-ray), in combination with community-based active case detection, expanded contact screening, and prompt treatment were the main strategies that contributed to this success [7]. Despite notable progress and investment to end TB, the country still lags behind in ensuring the large-scale implementation of these strategies [6, 8]. More than one-third of drug-susceptible TB (DS-TB) patients remain undiagnosed in Ethiopia; and this figure increases to 70% in cases of multidrug-resistant tuberculosis (MDR-TB) [9]. Furthermore, the high burden of TB coupled with the spread of human immunodeficiency virus (HIV) and MDR-TB poses further challenges to the health system. Addressing drug-resistant TB and comorbidities like HIV, diabetes, and malnutrition is crucial for enhancing treatment outcome [10].

One of the strategies to find the missed TB cases is through improved access to early diagnosis using molecular WHO recommended rapid diagnostic tests [3]. In Ethiopia, the diagnosis of TB is made using bacteriologic confirmatory techniques including microscopic examination, rapid molecular diagnostic tests such as X-pert MTB/RIF assay, Truenat and culture etc. In addition, supportive investigations (imaging techniques, histopathology, or biochemical analysis of fluids) assist clinicians in diagnosing smear-negative and extra-pulmonary TB cases [11].

The most recent national TB diagnostic algorithm recommends the scaling up of rapid diagnostic tests as the initial diagnostic test for all people with presumptive TB, unless the tests are not easily accessible [11]. The rapid molecular diagnostic tests that are in use in Ethiopia is predominantly GeneXpert (Xpert Ultra, Xpert MTB/XDR), but also includes Truenat, line probe assays (LPA), and urine lateral flow lipoarabinomannan (LF-LAM) [7]. In addition, the national algorithm includes X-ray for screening of TB to improve the yield of TB diagnostic tools [7, 11]. However, the country has low coverage of rapid TB diagnostic tests due to health-system resource constraints, including infrastructure, financing and human resources [12]. By 2020, only 10% of public health facilities have the capacity to do onsite rapid molecular diagnostic tests for TB, while additional 3500 health facilities are networked by a system for integrated specimen referral and result delivery [7]. As the country's TB diagnostics pipeline continues to expand, evaluating the potential impact and cost of diagnostic tools would help in prioritizing the most cost-effective intervention that ensures the optimal use of scarce resources. This study aims to evaluate the cost-effectiveness of TB diagnostics in Ethiopia through a modeling study.

## Methods

### Study population and design

The study population comprised an adult population aged 15 years with presumptive TB cases in Ethiopia. A presumptive TB case refers to an individual with symptoms or signs consistent with TB or with a chest X-ray abnormality suggestive of TB.

### Intervention

In Ethiopia, the diagnosis of TB is made using conventional TB diagnostic tools such as smear microscopy, drug susceptibility testing, or WHO approved rapid diagnostic tests like X-pert MTB/RIF, and Truenat. Additional supportive tests, including histopathological and radiologic examinations are employed to diagnose TB. Adhering to the national TB guideline and algorithms for TB diagnosis, the study evaluated various diagnostic tools or platforms: (i) X-pert MTB/RIF, (ii) Truenat, (iii) CXR screening followed by X-pert MTB/RIF, (iv) TB LAMP, and (v) smear microscopy.

### Model structure and assumptions

**Fig. 1:**
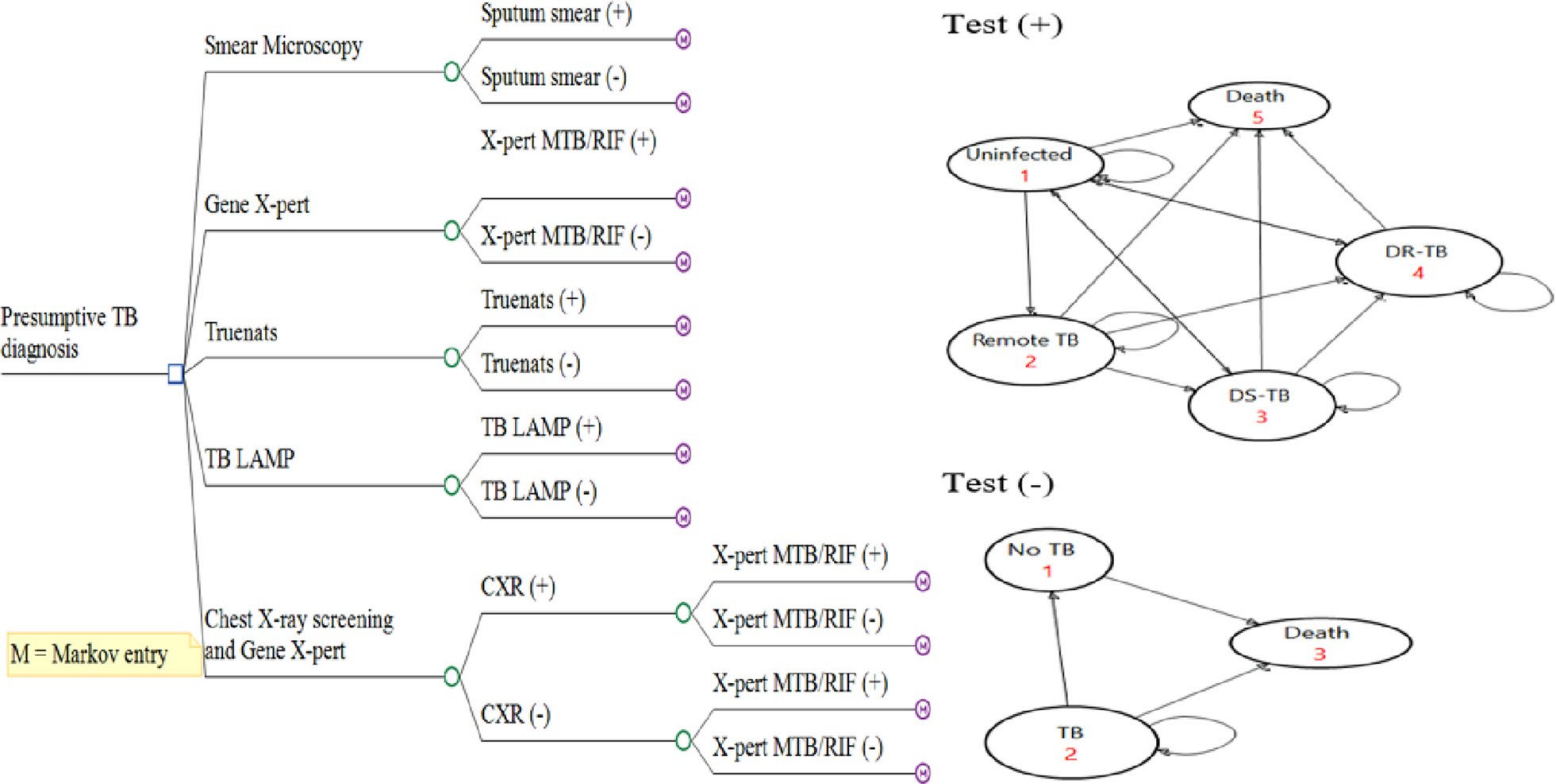
A hybrid Markov (decision and Markov) model structure.

A hybrid model composed of decision tree and Markov state transition was employed to estimate the cost-effectiveness of various TB diagnostic tools in a hypothetical adult cohort of presumptive TB cases aged 15 years old who were followed over a lifetime horizon (Fig. 1). A hybrid Markov model is an economic evaluation model that integrates a decision tree and a Markov model to assess the cost-effectiveness of interventions. The decision tree component captures discrete events or decisions within a short timeframe, while the Markov model extends the analysis over a more extended time horizon or life-time, which helps in predicting patient prognosis and estimating long-term effect and costs. Such a model provides a comprehensive framework for evaluating interventions by considering both immediate outcome as well as considers the evolving dynamics of patient states over a lifetime horizon [13].

In the decision tree, any presumptive TB cases would be examined with recommended TB diagnostic platforms, which indicates a person could either have a negative or positive laboratory test result. The TB test result is further stratified by the likelihood of TB disease conditional on the probability of receiving the test (i.e., positive, and negative predictive values), which are also dependent on the efficacy of the diagnostic methods (i.e., sensitivity and specificity) and incidence of TB. The presence or absence of TB disease among individuals with positive and negative test result will determine the starting probabilities of the Markov model health states.

The Markov model presents the patient pathways and long-term prognosis of a hypothetical cohort of adult populations followed over a lifetime. The model included five health states: un-infected with TB, remotely infected TB, drug-susceptible TB, drug resistant TB, and death. In the model, for a positive test branch, individuals in the un-infected health states can either stay in the same state or transit to other health states. Similarly, individuals with remote infection can be re-infected, re-activated or remain in the same state or can develop active TB (i.e., either drug-susceptible or drug-resistant TB) from reactivation and re-infection or die from the disease. Similarly, individuals in drug-susceptible and drug-resistant TB states would receive anti-TB treatment, which results in favorable and unfavorable treatment outcomes. Individuals in the negative test branch would either not have TB or undiagnosed TB diseases, which they could spontaneously get cured of, or they might have an ongoing illness or have died from the disease. The individual in the latter group would experience a higher probability of mortality. All individuals in each health state would also face a probability of dying from other causes and estimated based on the Ethiopian life tables (Fig. 1).

### Model parameters

This study pooled data from systematic review, global reports (i.e., WHO reports, life tables, global burden of disease) and in country published sources. The model input parameters used in this study are displayed in Table 1.

### Measurement of effectiveness and other key model inputs

The effectiveness of TB diagnostic platforms, sensitivity, and specificity of a test were pooled from systematic reviews and in-country published literature. The epidemiological and disease natural history parameters such as incidence, type of TB, infection rate, effective contact rate, reactivation rate, treatment success rate, risk of death, and spontaneous cure among untreated TB were sourced mainly from WHO and previously published sources (Table 1). The annual risk of TB infection was calculated from the incidence of smear positive, relative infectiousness of smear negative TB cases and effective contact rate [14]. The age specific mortality rates from TB and other causes death were extracted from the Global burden of disease result tool and WHO life table estimates (Ethiopia), respectively, in 2019. Although age-specific TB death rates were not stratified by drug resistance, drug resistance to any anti-TB treatment has been associated with greater mortality. To account for the higher mortality among drug-resistant cases, the age-specific TB mortality rate is raised by 5.5 [15].

**Table 1:** Input parameters used in the hybrid Markov (decision and Markov) model

Parameter	Value (range)	Distribution	References
Population	114,963,583	Point estimate	[16]
TB incidence per 100,000 population	132 (92–178)	Beta	[1]
Prevalence of TB among latently infected TB cases	0.167	Point estimate	[17]
Prevalence of MDR-TB among new cases (%)	2.18(1.44–2.92)	Beta	[18]
Prevalence of MDR-TB among previously treated cases (%)	12 (11–13)	Beta	[18]
Proportion of bacteriologically confirmed TB	0.62	Point estimate	[1]
Proportion of clinically diagnosed pulmonary negative and extra-pulmonary TB	0.38	Point estimate	[1]
Proportion of DS-TB (%)	0.99	Point estimate	[1]
Relative infectiousness of smear negative TB rate (95% CI)	0.22 (0.16–0.32)	Beta	[19]
Risk of death among MDR-TB as compared to DS-TB	5.55 (2.53–12.20)	Log-normal	[15]
Effective TB contact rate	17		[20, 21]
TB infection rate (force of infection)	0.016 (0.011–0.022)	Beta	Author’s cal. [1, 16, 19–21]
Reduction in the probability of rapid TB progression due to latent TB infection (95% Confidence Interval (CI))	0.79 (0.7–0.86)	Beta	[22]
Annual rate of reactivation from latent infection to active TB disease (95% CI)	0.0007 (0.00048–0.001)	Beta	[23]
Sensitivity of X-pert MTB/RIF (95% CI)	0.88 (0.84–0.92)	Beta	[24]
Specificity of X-pert MTB/RIF (95% CI)	0.99 (0.98–0.99)	Beta	[24]
Sensitivity of sputum smear microscopy (SSM) (95% CI)	0.50 (0.34–0.64)	Beta	[25]
Specificity of SSM (95% CI)	0.96 (0.85–0.99)	Beta	[25]
Sensitivity of Truenat (95% CI)	0.864 (0.67–0.95)	Beta	[26]
Specificity of Truenat (95% CI)	0.993 (0.95.8–0.99.9)	Beta	[26]
Sensitivity of X-pert MTB/RIF as an add-on after a negative SSM (95% CI)	0.68 (0.61–0.74)	Beta	[24]
Specificity of X-pert MTB/RIF as an add-on after a negative SSM (95% CI)	0.99 (0.98–0.99)	Beta	[24]
Sensitivity of chest X-ray as a screening tool (95% CI)	0.98	Point estimate	[27]
Specificity of chest X-ray as a screening tool (95% CI)	0.75	Point estimate	[27]
Sensitivity of X-pert MTB/RIF followed chest X-ray screen	0.9	Point estimate	[27]
Specificity of X-pert MTB/RIF followed chest X-ray screen	0.99	Point estimate	[27]
Sensitivity of TB-LAMP	0.803 (0.70–0.875)	Beta	[28]
Specificity of TB-LAMP	0.977 (0.96–0.987)	Beta	[28]
DS-TB treatment success rate (standard error (SE))	0.91 (0.37)	Beta	[29]
Proportion of DS-TB failure (SE)	0.01 (0.003)	Beta	[29]
Proportion of DS-TB death (SE)	Age specific	Interval estimate	[30]
MDR-TB treatment success rate (SE)	0.71 (0.11)	Beta	[29]
Proportion of MDR-TB failure (SE)	0.03 (0.01)	Beta	[29]
Proportion of DS-TB relapse after cure	0.01	Point estimate	Author’s
Proportion of DS-TB default cured	0.71	Point estimate	Author’s
Proportion of MDR-TB relapse after cure	0.4	Point estimate	Author’s
Proportion of untreated TB death (i.e., 5- and 10-year case fatality rate)	0.55, 0.72	Point estimate	[31]
Proportion of TB spontaneous cure	0.28	Point estimate	[32]
Probability of success with inappropriate treatment	0.48 (0.48–0.73)	Beta	[33]
Utilities*			
Un-infected	1	Beta	
Remotely infected (95% CI)	0.82 (0.80–0.85)		34, 35
DS-TB, median (Inter quartile range (IQR))	0.69 (0.57–0.77)		34, 35
Drug-resistant TB, median (IQR)	0.51 (0.39–0.73)		34, 35
Death	0		
Unit cost of SSM (SE)	3.4 (0.75)	Gamma	[12]
Unit cost of Chest X-ray	3.8 (1.9–7.6)	Gamma	[36]
Unit cost of X-pert MTB/RIF (SE)	13.1 (10.0)	Gamma	[12]
Unit cost of Truenat	13.2 (12.8–13.8)	Gamma	[37]
Unit cost of TB-LAMP (SE)	11 (2.2)	Gamma	Input from region
Unit cost of first line TB treatment	294.8 (265.5–323.6)	Gamma	[38]
Unit cost of second line TB treatment	2074.6 (1925.4–2223.6)	Gamma	[38]

*Disutility for each state is 1- utility value

### Measurement of health outcomes and costs

The primary outcome measures includes the cost per patient, the disability-adjusted life years (DALYs) accrued per patient, and incremental cost-effectiveness ratios (ICER) associated with each diagnostic tool. Utility weight of un-infected, remotely infected, drug-susceptible, and drug-resistant TB health states were obtained from published literature. Disutility values (i.e., 1- utility value) were used to estimate disability weight. The health system cost estimates for each diagnostic platform including TB and MDR-TB treatment were sourced from published literature (Table 1). Cost estimates were adjusted for the year 2019 using the consumer price index and reported in United States Dollar ($). All costs and health outcome measures were discounted by 3% annually.

### Analytic methods

The analysis was performed from the health system perspective, and the transition between the health states assumed to occur halfway through the annual time-step. The health status of adult cohort members who continued to survive each year at the start of the simulation was determined by what had happened the year before. The output of the model included costs, life-years, and DALY averted. The incremental cost-effectiveness ratio (ICER) was expressed as the ratio of incremental cost and DALY averted for the various TB diagnostic tools compared to the next best alternative. The ICER results are evaluated against the WHO-recommended willingness-to-pay threshold for Ethiopia (i.e., $856, equivalent to 1 times the GDP per capita) to assess the cost-effectiveness of the TB diagnostic tools. Both HIV/AIDs and TB mono-resistant cases were not taken into consideration in the modelling for ease of analysis.

### Sensitivity analysis

A sensitivity analysis was performed to check the robustness of the finding. A one-way sensitivity using tornado diagram was performed using the high and low values of input parameter to show the effect of each specific parameter on the estimated ICERs. The joint uncertainty of the model’s key parameters was assessed using probabilistic sensitivity analysis (PSA). Probabilistic sensitivity analysis was carried out using pre-defined distributions for each input parameter, and the result was produced with 10,000 simulations using Monte Carlo simulation. The PSA findings were plotted using a cost-effectiveness acceptability curve. The curve shows the degree to which a particular TB diagnostic strategy was the most cost-effective over a range of willingness to pay thresholds per DALY averted. In the PSA analysis, the standard error was recalculated using the mean and confidence interval for each distribution. In addition, for point estimates parameters, the standard error was adjusted to 20% of the mean. All analysis were performed using TreeAge pro-2022 software.

## Result

The estimated cost and effectiveness of each TB diagnostic strategies over lifetime are shown in Table 2. In the base-case, the discounted mean incremental cost of TB diagnosis and treatment ranges from $ 60 to 212. Similarly, the discounted DALY averted ranges from 0.1 to 0.78 per TB diagnosis and treatment, compared to the next best alternative. Among the diagnostic interventions considered, X-pert MTB/RIF is the most cost-effective diagnostic algorithm for TB, which resulted in an estimated ICER of $ 276 per DALY averted, compared to the next best alternative. The next optimal strategy is TB LAMP, with ICER of $ 274 per DALY averted. The combination of chest X-ray screening with X-pert MTB/RIF for TB diagnosis is found to be less cost-effective (i.e., < 3 times GDP per capita) as compared to X-pert MTB/RIF alone. In our analysis, the Truenat diagnostic platform had an ICER greater than that of a more effective intervention, which is X-pert MTB/RIF test and was extendedly dominated, indicating that a linear combination of TB-LAMP and X-pert MTB/RIF test would be a preferable strategy than using Truenat alone. This indicates that using the X-pert MTB/RIF test for a certain proportion of cases and the TB-LAMP test for others would provide more benefits at a lower cost than using the Truenat test.

**Table 2:** Estimated cost-effectiveness result of rapid diagnostic algorithms in Ethiopia

Strategy	Cost	Inc. cost	Effectiveness (DALY)	Inc. eff (DALY averted)	ICER ($/DALY averted)
Smear microscopy	109.9		7.98		
TB-LAMP	321.4	212.3	7.20	0.78	273.8
Truenat	381.2	59.8	7.02	0.19	ext. dom.*
X-pert MTB/RIF test	381.6	60.3	6.98	0.22	275.7
CXR screening followed by X-pert MTB/RIF test	483.1	101.4	6.92	0.06	1,665.8

*ext.dom* Extended Dominance

**Fig. 2:**
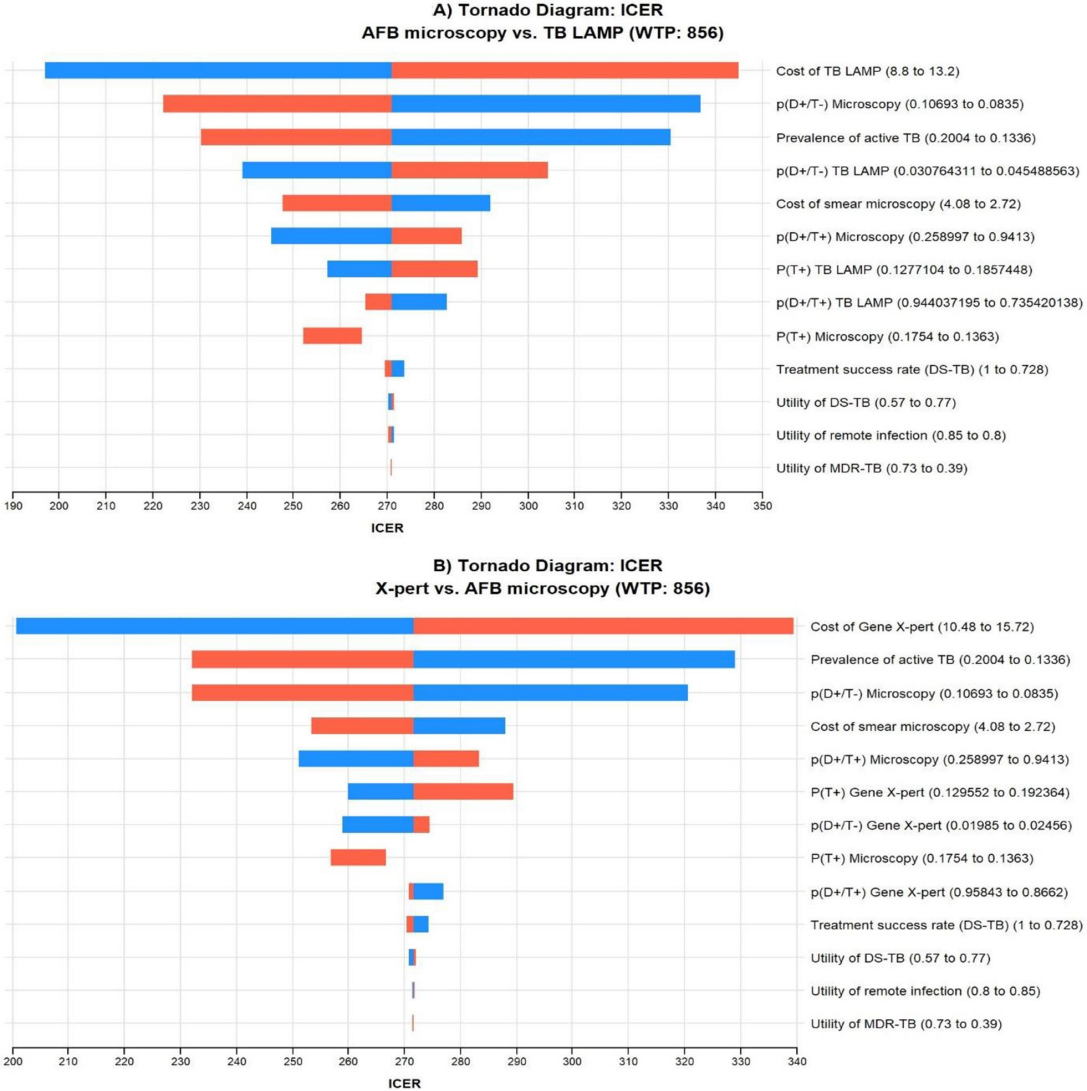
Tornado diagram of (2a) TB LAMP vs AFB microscopy and (2b) X-pert MTB/RIF vs AFB microscopy. Abbrevations: AFB: Acid- Fast Bacilli; DS-TB: Drug-susceptible TB; ICER: Incremental cost-effectiveness ratio; TB-LAMP: Loop-Mediated Isothermal Amplification for TB; MDR-TB: Multidrug-resistant TB; p(T+): Probability of test positive; p(D+/T-): probability of TB disease conditional on test negative; TB: Tuberculosis; p(D+/T+): Positive Predictive Value; WTP: Willingness to Pay.

The tornado diagram in Fig. 2 demonstrates the influence of varying key input parameters on the base-case result. In Fig. 2a the cost of TB-LAMP, the probability of TB positivity among negative microscopy test results, and the prevalence of active TB were the main parameters influencing the cost-effectiveness results. An increase in the unit cost of TB-LAMP elevated the ICER value, making it less attractive; conversely, the decline in the probability of TB positivity among negative microscopy tests, and prevalence of active TB decreased the ICER value, rendering it more attractive, though not surpassing the WTP threshold. Similarly, in Fig. 2b the cost of X-pert MTB/RIF proved to be the most influential variable, followed by the prevalence of active TB and the probability of TB positivity among negative microscopy test results. Other important variables influencing the ICER value include the probability of a positive test, positive predictive value, and treatment success rate.

**Fig. 3:**
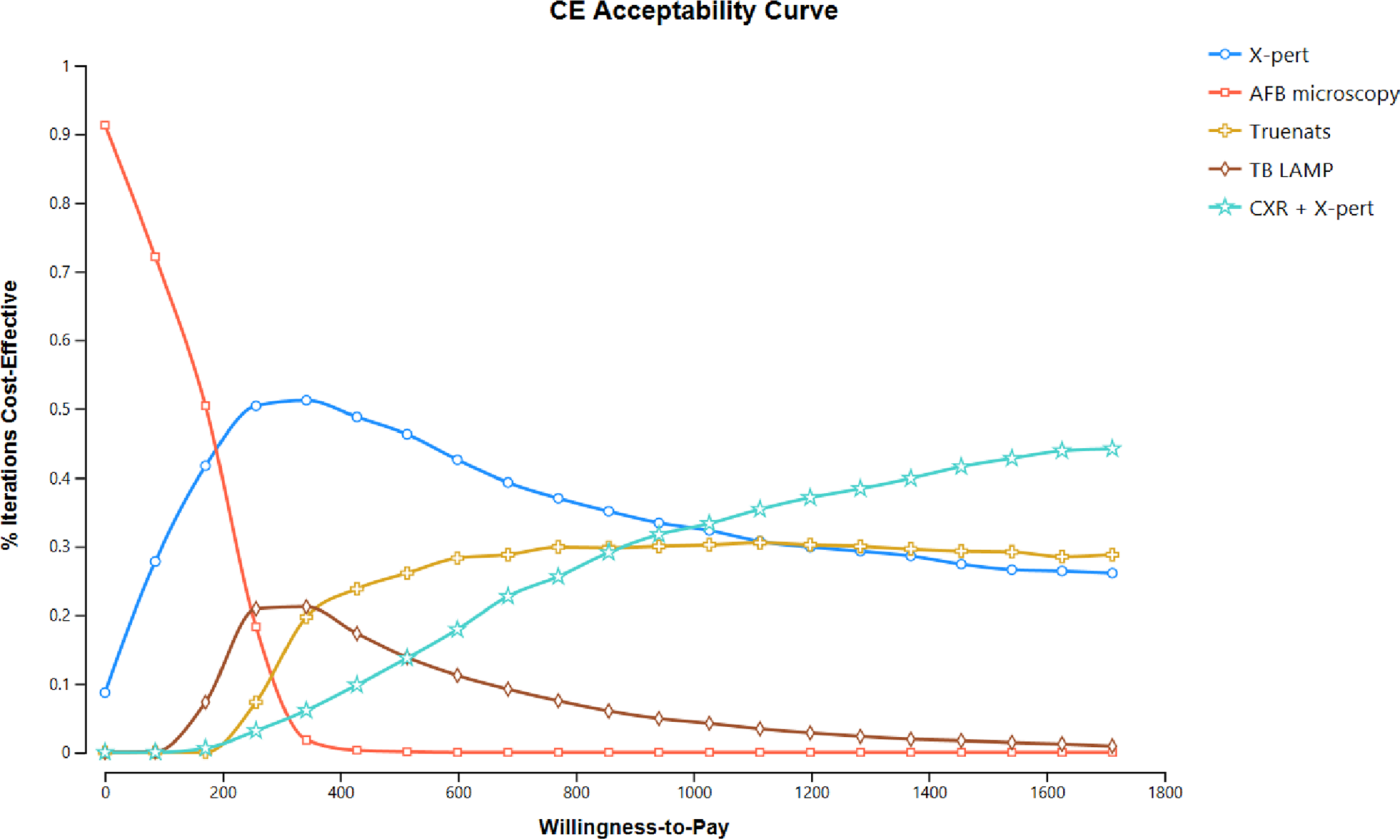
Cost-effectiveness acceptability curve of TB diagnostic tools using 10,000 iterations of Monte Carlo-simulation at various level of willingness to pay threshold.

The probabilistic sensitivity analysis incorporated key input parameters in Monte Carlo simulations (Fig. 3). At a willingness-to-pay threshold of $856 per DALY averted (equivalent to 1 × GDP per capita), X-pert MTB/RIF had the highest net benefit 35% of the time. A combination of chest X-ray and X-pert MTB/RIF accounted for 29% of the net benefit, while TB-LAMP contributed to 6% of the net benefit.

The TB detection rate is increased by one-fifth with rapid TB diagnostic tools, as indicated in Table 3 below. The X-pert MTB/RIF test would avert additional 10,000 DALYs at a total cost of $3,816,000 as compared to smear microscopy for a cohort of TB patients followed over lifetime.

**Table 3:** Population and health system effect of TB diagnostic tools for a cohort of 10,000 TB patients in Ethiopia

Strategy	Percent increase in TB detection	DALY per cohort	DALY averted per cohort	Total budget ($) per cohort
Smear microscopy	Ref	79, 800	Ref	1,099,000
TB-LAMP	15.7%	72,000	7800	3,214,000
Truenat	22.2%	70,200	9600	3,812,000
X-pert MTB/RIF	21.2%	69,800	10,000	3,816,000
CXR screening followed by X-pert MTB/RIF	23.0%	69,200	10,600	4,831,000

## Discussion

The TB program funding is distributed across a range of key TB strategies, such as prevention, case finding, diagnostics, and treatment. The expansion of a specific strategy will divert scarce resources from competing but more cost-effective strategies of the TB program. There are various laboratory diagnostic tools used for the diagnosis of TB in Ethiopia. Prioritising the most cost-effective diagnostic tools is essential for efficient allocation and utilization of available resources. Therefore, economic evaluation is useful in informing scale-up decisions by highlighting which diagnostic tests are cost-effective in contrast to other alternative TB diagnostic tools.

In this study, a hybrid Markov state transition model was used to evaluate the cost-effectiveness of selected TB diagnostic tools in the country. The study indicated that the use of X-pert MTB/RIF would be the most cost-effective strategy with the greatest number of DALYs averted compared to other TB diagnostic tools. The benefits of this strategy stem from improved sensitivity and specificity of the test when compared to other TB diagnostic tools. Our finding is in line with several studies that assessed the cost-effectiveness of TB diagnostic tests in sub-Saharan African countries, including Ethiopia, and found that the selected diagnostic platform to be highly cost-effective [12, 36, 39]. For instance, the current study base-case result for the X-pert MTB/RIF assay as a primary diagnostic test indicated an ICER of $274 per DALY averted, in contrast to previous studies’ findings of an ICER of $5 per DALY averted and $20 per TB case diagnosed, respectively. While the studies highlighted the cost-effectiveness of the X-pert MTB/RIF assay, the variations in estimated values might be attributed to the differences in modelling approaches and other factors. In addition, the prior studies used a decision analysis framework with a shorter time horizon, our research employed a Markov model with a lifetime horizon, capturing a distinct sequence of events aligning with the natural history of TB disease. Additionally, there are variations in the choice of outcome measures and comparators, discounting, and disaggregation by HIV status [12, 36]. However, our results are more comparable with another study conducted in Ethiopia, which found that the ICER of the rollout of X-pert MTB/RIF test would be $ 370 per DALY averted [40].

Likewise, using a chest X-ray to prioritize presumptive TB cases for X-pert MTB/RIF test is less likely to be cost-effective at a WTP threshold of one times GDP per capita ($856). Chest X-rays can be used to prioritize individuals for bacteriological confirmation (such as X-pert MTB/RIF), which can lead to early detection and treatment of TB. However, the cost-effectiveness of the strategy, specifically Chest X-ray screening followed by X-pert MTB/RIF, remains doubtful unless the country is willing to allocate three times the GDP per capita per DALY averted. This raises concerns about the affordability of this strategy, emphasizing the need for a detailed analysis of resource implications in developing settings to ensure its wider implementation alongside other diagnostic tools. The finding is also supported by a study carried out in a referral hospital that revealed the addition of Chest X-ray is unlikely to substantially improve the diagnostic accuracy and case finding of TB [41]. Accordingly, a prior study carried out in a high TB burden setting indicates the use of chest X-ray to screen presumptive TB cases is unlikely to be cost-effective [39].

The cost-effectiveness result is mainly influenced by several factors, including prevalence of active TB, the cost of diagnostic tests, and the likelihood of TB cases among negative tests. In X-pert MTB/RIF and TB- LAMP tests, highly cost-effective tests as compared to microscopy, increasing the prevalence of presumptive TB population will always result in the reduction of ICER from the baseline. This result signifies the effectiveness of the two tests increases with a higher prevalence of TB. Increased costs of these tests would increase the ICER from the baseline, but an increase in the probability of TB cases among negative tests of the comparator group (i.e., microscopy, base-case) would result in a decrease in the ICER.

The main limitations of this study arise from reliance on published sources for key input parameters (i.e., prevalence, probabilities, costs, etc.). Although we made efforts to mitigate parameter input uncertainty through sensitivity analysis, relying on such sources affects the accuracy of the findings. In addition, all the diagnostic platforms in the national TB guideline are not included in this study due to a lack of sufficient data and their limited implementation. For instance, LF-LAM is recommended for diagnosing TB in individuals with advanced HIV disease, however this represents a very small percentage of the target population (i.e., a 3.9% TB/HIV co-infection rate). Similarly, cytological diagnostic tests, are available in selected tertiary care centres, are largely inaccessible for supporting extra-pulmonary TB diagnosis and are excluded from our study. Even though the suggested diagnostic tools are effective, they pose challenges when dealing with extraordinary specimen and paediatric cases as they require large volumes of specimens for optimal performance. Additionally, the sensitivity of these tools in detecting TB may be compromised in critically ill patients [42, 43]. Recognizing the limitations in the proposed diagnostic tools necessity the need to investigate various diagnostic tools including imaging TB diagnostics tailored to diverse population groups, including paediatric cases, patients with extra-pulmonary TB, and severe clinical conditions. Lastly, while multi-disease integrated testing has the potential to improve the efficiency of rapid TB diagnostic tools in specific contexts, its feasibility and implementation require careful assessment of local factors and benefit–cost analysis. Despite these limitations, the study attempted to capture the natural history and long-term outcomes of TB patients by including information on TB transmission, progression, diagnosis and treatment, as well as mortality and costs. The study findings suggest that the scale-up of WHO-approved rapid diagnostics, such as the X-pert MTB/RIF assay can improve case detection and prompt treatment of TB diseases. It also underscores the importance of making strategic investments in cost-effective diagnostic technologies to effectively address the TB burden in resource-limited settings.

## Conclusion

The X-pert MTB/RIF assay is the most cost-effective diagnostic strategy in high burden countries like Ethiopia. The routine use of the X-pert MTB/RIF test as the primary diagnostic test has a beneficial effect on the control of TB disease in the country. Furthermore, a placement strategy based on local TB burden could optimize its utilization and further minimize operational costs.

## Data Availability

The datasets used and/or analyzed during the current study are available from the corresponding author upon reasonable request.

## Abbreviations

CI: Confidence Interval
DALY: Disability-adjusted life years
GDP: Gross domestic product
ICER: Incremental cost-effectiveness ratio
IQR: Inter quartile range
HIV: Human immunodeficiency virus
MTB/RIF: Mycobacterium tuberculosis complex (MTBC) and resistance to rifampin (RIF)
LED: Light-emitting diodes
PSA: Probabilistic sensitivity analysis
SE: Standard error
SSM: Sputum smear microscopy
TB: Tuberculosis
WHO: World Health Organization
